# Hesperidin alleviates insulin resistance by improving HG-induced oxidative stress and mitochondrial dysfunction by restoring miR-149

**DOI:** 10.1186/s13098-021-00664-1

**Published:** 2021-04-29

**Authors:** Miao Tian, Yu-Bo Han, Cheng-Cheng Zhao, Li Liu, Fu-Li Zhang

**Affiliations:** 1 Heilongjiang University of Chinese Medicine, Harbin, 150040 Heilongjiang People’s Republic of China; 2 The First Department of Cardiovascular, First Affiliated Hospital, Heilongjiang University of Chinese Medicine, No. 26 Heping Road , Xiangfang District, Harbin, 150040 Heilongjiang People’s Republic of China; 3 School of Basic Medicine, Heilongjiang University of Chinese Medicine, Harbin, 150040 Heilongjiang People’s Republic of China

**Keywords:** Hesperidin, Diabetes, miR-149, Mitochondrial dysfunction, Insulin resistance

## Abstract

**Background:**

Hesperidin, a natural flavanone, has been proven to have multiple protective effects in diabetic rats, such as antioxidant, anti-inflammatory and anti-apoptotic effects. However, the molecular mechanisms underlying the effects of hesperidin are not well elucidated.

**Methods:**

LO2 cells were stimulated with high glucose (HG, 33 mM) for 24 h to establish a model of oxidative stress. Then, cell viability was determined using the MTT assay. The antioxidant activities, including the reactive oxygen species (ROS), malondialdehyde (MDA), superoxide dismutase (SOD) and glutathione peroxidase (GPx) levels, mitochondrial membrane potential (MMP) and adenosine-triphosphate (ATP) production, were measured with the corresponding kits. The levels of gene expression, protein expression and methylation were detected using qRT-PCR, western blotting and methylation-specific PCR (MSP) assays, respectively.

**Results:**

Compared to the NG treatment, hesperidin treatment increased the viability and improved the oxidative stress, mitochondrial dysfunction and insulin resistance of HG-treated LO2 cells, and these effects were correlated with heightened SOD and GPx activities, increased MMP level and ATP generation, reduced MDA, ROS and glucose levels, and activated GSK3β/AKT and inactivated IRS1 signals. Mechanistically, hesperidin treatment enhanced the miR-149 expression level by reducing its promoter methylation by inhibiting DNMT1. Importantly, knockdown of miR-149 obviously abolished the biological roles of hesperidin.

**Conclusions:**

Our findings demonstrated that hesperidin treatment ameliorated HG-induced insulin resistance by reducing oxidative stress and mitochondrial dysfunction partly by suppressing DNMT1-mediated miR-149 silencing.

## Background

Diabetes is a group of lifelong metabolic diseases characterized by chronic hyperglycaemia, which is caused by multiple factors [1]. The worldwide prevalence of diabetes was approximately 347 million in 2008, and this incidence is expected to approximately triple by 2050. The high blood sugar level of patients with diabetes can increase oxidative stress [2–4], induce mitochondrial dysfunction [5, 6], disrupt intracellular redox balance, and cause lipid peroxidation and insulin resistance, thereby leading to multiple complications, such as macrovascular and microvascular diseases and neuropathy [7–10].

Hesperidin is a type of flavonoid glycoside compound found in abundant levels in citrus fruits. Hesperidin has analgesic, antihypertensive, diuretic, hypolipidemic, anti-inflammatory, anti-cancer and anti-oxidant effects [11, 12]. Related literature indicates that hesperidin is considered to be a potential drug that can be used to treat diabetes and its related complications [13–15]. Hesperidin has shown anti-hyperglycemic, anti-hyperlipidemic, and anti-oxidant effects in rats with streptozotocin-induced diabetes [16], and could capable of suppressing retinal oxidative stress, neuroinflammation and apoptosis in diabetic rats [14]. However, the relevant underlying mechanism is still unclear. Therefore, it is of great research value to elucidate the mechanism by which hesperidin regulates the physiological processes of liver cells under high glucose conditions.

MicroRNAs (miRNAs) are single-stranded, noncoding RNA molecules with lengths ranging from 18 to 25 nucleotides and are encoded by endogenous genes [17]. miRNAs are critical regulators of multiple biological processes, such as apoptosis, oxidative stress, and inflammatory factors and are involved in the onset and development of diabetes [18, 19]. The roles of miRNAs in type-2 diabetes were first elucidated by Poy et al., who showed the important roles of miR-375 in insulin secretion [20]. Later, accumulating studies further confirmed the regulatory roles of miRNAs, such as miR-376, miR-375, miR-9, and miR-15a/b, in physiological processes of diabetic models [18, 21]. miR-149, which is highly related to multiple diseases, was downregulated in a HG-treated pancreatic beta cell line, and its overexpression markedly reduced ROS production and cell apoptosis, and elevated cell viability and insulin secretion [22]. Additionally, Zheng et al. also confirmed that miR-149 was decreased in insulin-resistant skeletal muscle cells and involved in the regulation of glucose uptake, mitochondrial dysfunction and insulin resistance-related signalling [23]. However, the mechanism underlying the function of miR-149 remains to be further explored.

This study intends to explore the effects of hesperidin on oxidative stress-induced mitochondrial dysfunction and insulin resistance in HG-treated LO2 cells, as well as the underlying mechanisms. The data will provide new evidences for the clinical application of hesperidin in diabetes.

## Materials and methods

### Cell Culture and treatments

Human normal hepatocytes (LO2 cells) were purchased from the Institute of Basic Medical Science, Chinese Academy of Medical Science (Beijing, China). The cells were grown in Dulbecco’s modified Eagle medium (DMEM) (Macgene Biotech) supplemented with 100 U/ml penicillin, 100 µg/ml streptomycin (Thermo Fisher Scientific) and 10% foetal bovine serum (FBS, Gibco) in a humidified incubator at 37 °C under 5% CO_2_.

To establish a diabatic model of diabetic oxidative stress in vitro as described in a previous study [24], LO2 cells were maintained in high-glucose (HG, 33 mM) medium for 48 h as HG group. LO2 cells cultured in normal glucose (NG, 5.5 mM) medium were used as the NG group. A series of hesperidin solutions with different concentrations (Shenggong, Shanghai, China) were prepared, and the cytotoxic effects were detected using the MTT assay.

### Construction of the expression vector and cell transfection

Small interfering RNA (siRNA) against DNMT1, miR-149 inhibitor and the corresponding negative controls, including siNC and inhibitor NC, were synthesized by GenePharma (Shanghai, China). In the current work, LO2 cells (4 × 10^5^ cells/per well) were maintained in 6-well plates, and then, the miR-149 inhibitor and si-DNMT1 or their corresponding controls (siNC or inhibitor NC) were transiently transfected into the cells using Lipofectamine 2000 (Invitrogen) according to the manufacturers’ instructions. After transfection for 24 h, the transfection efficiencies were determined using qRT-PCR.

### MTT assay

Cell viability was assessed using the MTT assay. Cells were seeded in 96-well plates at a density of 5 × 10^3^ cells/well. After treatment, MTT reagent (20 μL) was added to each well and incubated for 4 h. Then, 150 μL DMSO was added to each well and shaken 10 min. Then, the absorbance was measured at 490 nm. The OD value for the control cultures was considered as 100% viability, and the viability in the other samples is expressed as a percentage of the viability in the control group.

### Anti-oxidant enzyme assay

The antioxidant activity of the cells in this work was assessed based on the level of MDA and the enzyme activities of SOD and GPx. In brief, the MDA levels and SOD and GPx activities were analysed using a Lipid Peroxidation (MDA) Assay Kit (Sigma-Aldrich, St. Louis, MO), SOD Determination Kit (Sigma-Aldrich) and Glutathione Peroxidase Cellular Activity Assay Kit (Sigma-Aldrich), and the procedures were conducted according to the corresponding manufacturer’s instructions. The absorbances were measured using a microplate spectrophotometer (BioTek, USA).

### Glucose content assay

LO2 cells were cultured in 24-well plates (2 × 10^5^ cells/well) for 24 h. After the different treatments, the cells in each group were collected and washed twice with PBS. Next, the cells were placed in serum-free DMEM containing 25 mmol/L d-glucose and 1 nmol/L insulin and incubated for 3 h. The culture medium was collected to detect the glucose content using a glucose oxidase–peroxidase kit (GOD-POD kit, Abcam, Cambridge, UK). The absorbance was tested at 505 nm using a microplate spectrophotometer (BioTek, USA).

### ROS detection

The intracellular ROS level was determined using a MitoSOX™ Red mitochondrial superoxide indicator (Thermo Fisher Scientific, Invitrogen), a cationic derivative of dihydroethidium, to selectively examine the mitochondrial superoxide levels of live cells. In brief, cells were incubated with 5 μM mitoSOX™ red reagent working solution in the dark at 37 °C for 30 min according to the manufacturer’s procedure. Then, the cells were washed 3 times with warm buffer. Then, the ROS signals were imaged with a fluorescence microscope (C1-T-SM, Nikon, Japan), and the fluorescence intensity was quantified using ImageJ software.

### Mitochondrial membrane potential (MMP) detection

The level of the mitochondrial membrane potential (MMP) in cells was evaluated using JC-1 dye (Thermo Fisher Scientific, Invitrogen). In brief, the cells were collected and washed with PBS and incubated with JC-1 dye (5 mM) at 37 °C for 30 min. Then, the cells were washed and resuspended in PBS. Finally, the cells were imaged by a fluorescence microscope (C1-T-SM, Nikon, Japan), and the fluorescence intensity was analysed using Image J software.

### ATP assay

The ATP level was determined with an ATP Assay Kit (Beyotime, Nantong, China) following the manufacturer’s procedure. In brief, the cells were lysed in ice-cold ATP-releasing buffer and centrifuged at 12,000*g* for 5 min. The supernatant was transferred to 96-well plates and mixed with an equal volume of ATP detection working dilution. Then, the ATP content was analysed using a microplate spectrophotometer (BioTek, USA).

### Quantitative real-time PCR (qRT-PCR)

Total RNA was isolated from LO2 cells with TRIzol (Invitrogen, California, USA) and reverse transcribed into cDNA using the PrimeScript™ RT reagent Kit with gDNA Eraser (TaKaRa, Dalian, China). Then, qRT-PCR experiments were conducted with SYBR^®^ Premix Ex Taq™ II (TaKaRa) in an ABI 7500 system (Applied Biosystems, CA, USA). U6 or GAPDH was used as internal control to normalize the expression of the miRNAs or mRNAs, respectively. The data were calculated using the 2^−ΔΔCt^ method. The primer sequences were listed as Table 1.

**Table 1:** The Primer sequences

Name	Sequence
DNMT1 F	5′-GTTCCTCCTTCTGCCATCAAT-3′
DNMT1 R	5′-CGTCTCATCATCGTCCTTAGC-3′
GLUT2 F	5′-GTTCATGGTGGCCGAGTT-3′
GLUT2 R	5′-ATTGCGGGTCCAGTTGC-3′
GLUT4 F	5′-GACTCTGGGTGAAAGGG-3′
GLUT4 R	5′-GGGAAGGCTGAGTGAGA-3′
IRS1 F	5′- AGCACCTGGTGGCTCTACA-3′
IRS1 R	5′- CAGCTGCAGAAGAGCCTGGTA-3′
miR-149 F	5′-CGTCTGGCTCCGTGTCTTC-3′
miR-149 R	5′-GTCGTATCCAGTGCAGGGTCCGAGGTATTCGCACTGG ATACGACGGGAGT-3′
GAPDH F	5′-GAAGGGCATCTTGGGCTACAC-3’
GAPDH R	5′-GTTGTCATTGAGAGCAATGCCA-3’
U6 F	5′-CTCGCTTCGGCAGCACA-3’
U6 R	5′-AACGCT TCACGAATTTGCGT-3’

### MSP assay

Cell genomic DNA was extracted using a DNeasy tissue kit (Qiagen, Hilden, Germany). 5 μg genomic DNA was modified with bisulfite using an EpiTect Bisulfite kit (Qiagen). Then, the methylation level of the miR-149 promoter was detected using the following primers. Unmethylated primers (product size: 111 bp): (F) 5′-GAGTTTTGTAGAAGGAAGTTAGTGG-3′, (R) 5′-AAAACCTCAAACAAACTAAATCAAA-3′; methylated primers (product size: 108 bp): (F) 5′-GGAGTTTCGTAGAAGGAAGTTAGC-3′, (R) 5′-TAAAAACCTCGAACAAACTAAATCG3′. The reaction products were detected by 2% agarose gel electrophoresis.

### Western blotting

LO2 cells were lysed in RIPA buffer (Sigma) on ice and then centrifuged at 12,500 rpm for at least 15 min. The concentrations of the total proteins were quantified using a BCA protein assay kit (Sigma). Next, equal amounts of the protein samples were separated by sodium dodecyl sulfate–polyacrylamide gel electrophoresis (SDS-PAGE) and then transferred onto PVDF membranes (Sigma). Then, the proteins on the PVDF membranes were stained with Ponceau red and cut according to the corresponding molecular weight. After blocking in TBS buffer containing 5% non-fat dry milk for 1 h at room temperature, the clipped membranes were incubated with primary antibodies and softly shaken overnight at 4 °C. After washing and removing the unbound primary antibodies, the membranes were incubated with the secondary horseradish peroxidase (HRP)-labelled antibodies for 1 h at room temperature. Finally, the blots were detected and visualized using an ECL reagent (Bio-Rad). The intensity of the blots was quantified using ImageJ software. All the primary antibodies, including DNMT1 (1:1000), p-GSK3β (1:1000), GSK3β (1:5000), p-AKT (1:500), AKT (1:500), p-IRS1 (1:1000), IRS1 (1:1000) and GAPDH (1/2500), and the secondary antibodies (1:1000) were purchased from Abcam.

### Statistical analysis

Each experiment in this work was repeated 3 independent times. GraphPad Prism 5.0 was used to statistically analyse all the data using t tests or ANOVA. All the data were presented as the Mean ± S. D., and a P value less than 0.05 was considered statistically significant.

## Results

### Hesperidin increased the viability of HG-treated LO2 cells.

**Fig. 1:**
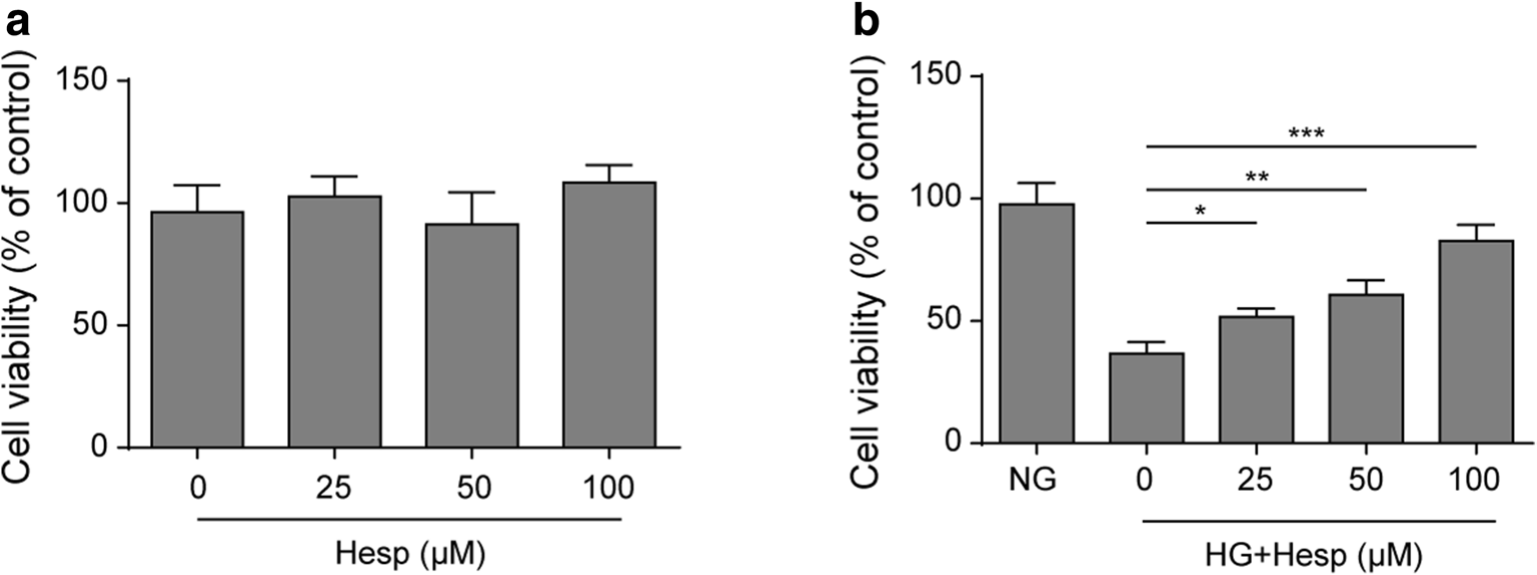
Hesperidin increased the viability of HG-treated LO2 cells.** a** The effect of hesperidin on LO2 cell viability was detected by MTT analysis. **b** The effect of hesperidin on the viability of HG-treated LO2 cells was detected by MTT analysis. All the data are presented as the mean ± S.D. *p < 0.05, **p < 0.01, *** *p* < 0.001

Hesperidin was proven to play a variety of beneficial effects on rats with diabetes [14]. Here, we first examined the toxic effect of hesperidin on LO2 cells. As described, different concentrations of hesperidin had no significant effect on the viability of LO2 cells, indicating that hesperidin had no toxic effect on LO2 cells (Fig. 1a). Then, we detected the effects of hesperidin on an HG-induced cell model. Compared to that of the NG group, the viability of the HG-induced cell group was significantly impaired, which was alleviated by hesperidin treatment in a concentration-dependent manner (Fig. 1b).

### Hesperidin improved oxidative stress and mitochondrial dysfunction in HG-treated LO2 cells.

**Fig. 2:**
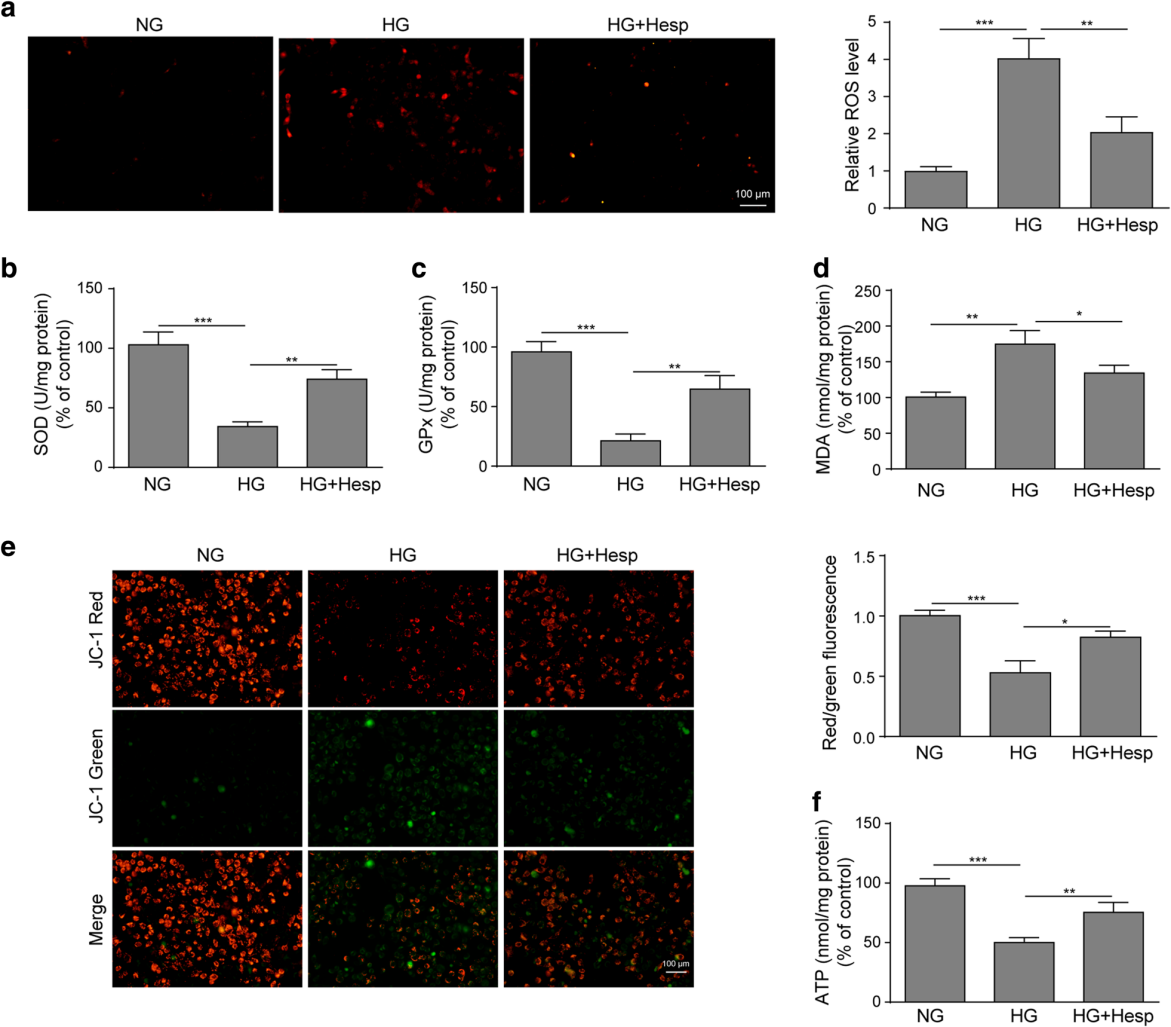
Hesperidin improved reduced oxidative stress and mitochondrial dysfunction in HG-treated LO2 cells. **a** The effect of hesperidin on ROS production was detected by a fluorescent MitoSox probe. **b–d** The effects of hesperidin on the levels of SOD, GPx and MDA were calculated by corresponding detection kits. **e** The effect of hesperidin on MMP was detected by JC-1 staining. **f** The effect of hesperidin on the ATP content was determined. All the data were presented as the mean ± S.D. *p < 0.05, **p < 0.01, *** *p* < 0.001

To further explore the effects of hesperidin on oxidative stress and mitochondrial function in diabetes in vitro, we determined the antioxidant activity, MMP level and ATP production. Figure 2a showed that the ROS level was markedly elevated in HG-treated LO2 cells, while hesperidin treatment clearly reduced the excessive level of ROS induced by HG. Similarly, the activities of SOD and GPx in HG-induced LO2 cells were markedly suppressed, and these levels were dramatically enhanced after hesperidin treatment (Fig. 2b, c). In contrast, the increased MDA content in HG-treated LO2 cells was significantly reduced by hesperidin treatment (Fig. 2d). In addition, the MMP level was showed a much lower level in HG-treated LO2 cells than in NG-treated LO2 cells, and hesperidin treatment obviously increased the MMP level in HG-treated LO2 cells (Fig. 2e). Similarly, hesperidin treatment markedly ameliorated the reduction of ATP level in HG-treated LO2 cells (Fig. 2f). In summary, these findings suggested that hesperidin improved the mitochondrial dysfunction of HG-treated LO2 cells by reducing oxidative stress.

### Hesperidin attenuated HG-induced LO2 insulin resistance.

**Fig. 3:**
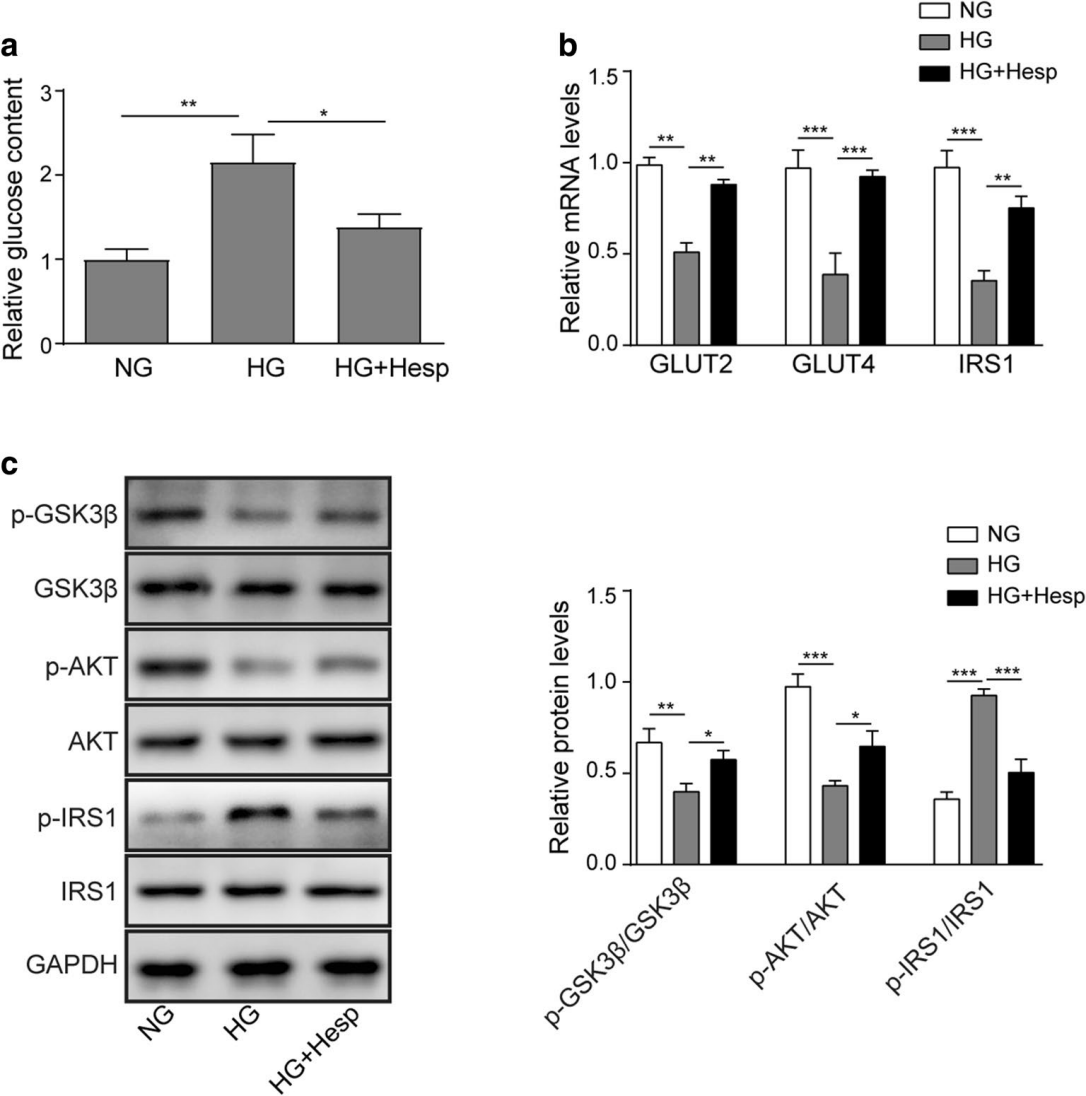
Hesperidin attenuated insulin resistance in HG-treated LO2 cells. **a** The effect of hesperidin on the glucose content in the culture medium of HG-treated LO2 cells was examined. **b** The effects of hesperidin on the expression of GLUT2, GLUT4 and IRS1 were detected by qRT-PCR. **c** The effects of hesperidin on the phosphorylation of GSK3β, AKT and IRS1 were quantified by western blotting. All the data were presented as the mean ± S.D. *p < 0.05, **p < 0.01, *** *p* < 0.001

It has been reported that oxidative stress and mitochondrial dysfunction are the main factors that cause insulin resistance, which a typical character is the elevated glucose content [24, 25]. As shown in Fig. 3a, compared to that in the NG group, the glucose level in the culture medium of the HG-treated LO2 cells was markedly increased. After hesperidin treatment, the glucose level was dramatically decreased. Subsequently, we further examined the expressions of glucose transport- and glycogen synthesis-related genes and signals. Figure 3b presented that the mRNA levels of GLUT2, GLUT4 and IRS1 were markedly suppressed in HG-treated LO2 cells, while the inhibition was markedly diminished by hesperidin treatment. A similar trend was observed by western blotting. Hesperidin treatment significantly weakened the HG-mediated inhibitory effects on GSK3β and AKT phosphorylation and the stimulatory effect on IRS1 phosphorylation (Fig. 3c). All of the above results suggested that hesperidin attenuated HG-induced LO2 insulin resistance.

### Hesperidin upregulated miR-149 expression by inhibiting DNMT1.

**Fig. 4:**
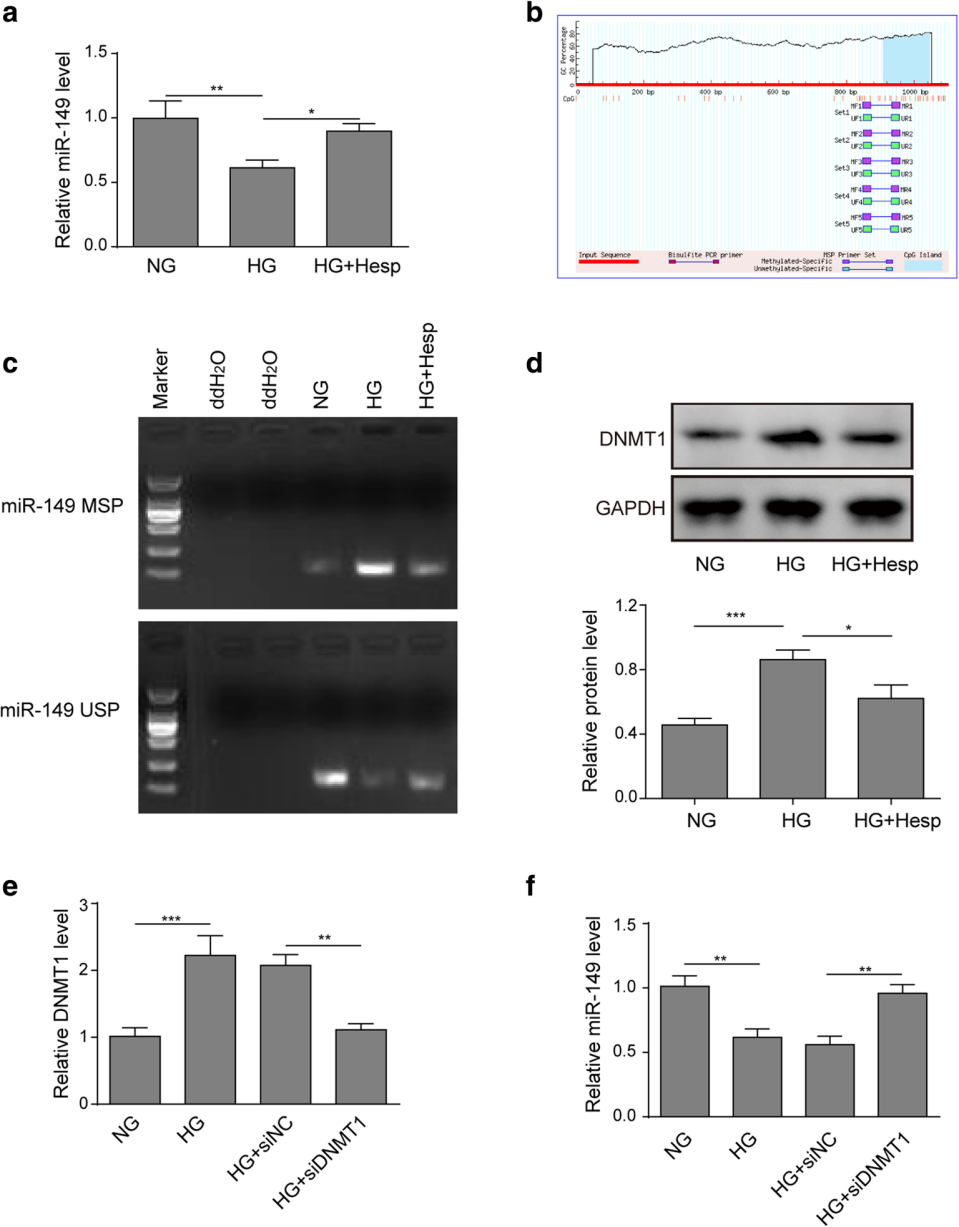
Hesperidin upregulated miR-149 expression by inhibiting DNMT1. **a** The effect of hesperidin on the expression of miR-149 was detected by qRT-PCR. **b** The CpG site of miR-149 in the promoter region (1000 to − 100 bp) was analysed by MethPrimer software (http://www.urogene.org/cgi-bin/methprimer/methprimer.cgi). **c** The effect of hesperidin on the methylation level of the miR-149 promoter was analysed by MSP assay. **d** The effect of hesperidin on the protein level of DNMT1 was determined by western blotting. **e** Transfection efficiency of the siDNMT1 plasmids was detected by qRT-PCR. **f** The effect of DNMT1 knockdown on the expression of miR-149 was detected by qRT-PCR. All the data were presented as the mean ± S.D. *p < 0.05, **p < 0.01, *** *p* < 0.001

To further reveal the underlying mechanism of the effects of hesperidin, we tried to identify the downstream functional molecules. Noticeably, hesperidin is a flavonoid glycoside that was identified as a DNA hypomethylating agent that modulates the epigenome, thus leading to changes in gene expression patterns [26, 27]. Preliminary analysis found that miR-149 was an important regulator of insulin resistance and mitochondrial dysfunctions, and that its expression was regulated by promoter CpG methylation [23, 28]. As demonstrated in Fig. 4a, compared with that in the NG group, miR-149 was downregulated in the HG-treated LO2 cell group. Prediction analysis (MethPrimer) showed that the promoter of miR-149 (1000 to − 100 bp) contained a CpG locus (Fig. 4b). Consistently, the MSP assay results showed that the methylation level of the miR-149 promoter was higher in HG-treated LO2 cells. After hesperidin treatment, the methylation level of the miR-149 promoter was markedly reduced (Fig. 4c). DNMT1, a main DNA methyltransferase, is responsible for maintaining the pattern of DNA methylation. Here, western blotting assay demonstrated that HG treatment significantly increased DNMT1 expression, while its was obviously decreased after hesperidin treatment (Fig. 4d). qRT-PCR assay demonstrated that si-DNMT1 plasmids markedly reduced the excessive mRNA level of DNMT1 in HG-treated LO2 cells (Fig. 4e). As expected, DNMT1 downregulation significantly elevated miR-149 expression in HG-treated LO2 cells (Fig. 4f). These data indicated that hesperidin promoted miR-149 expression by reducing DNMT1-mediated promoter methylation level.

### Knockdown of miR-149 partly reversed the biological roles of hesperidin.

**Fig. 5:**
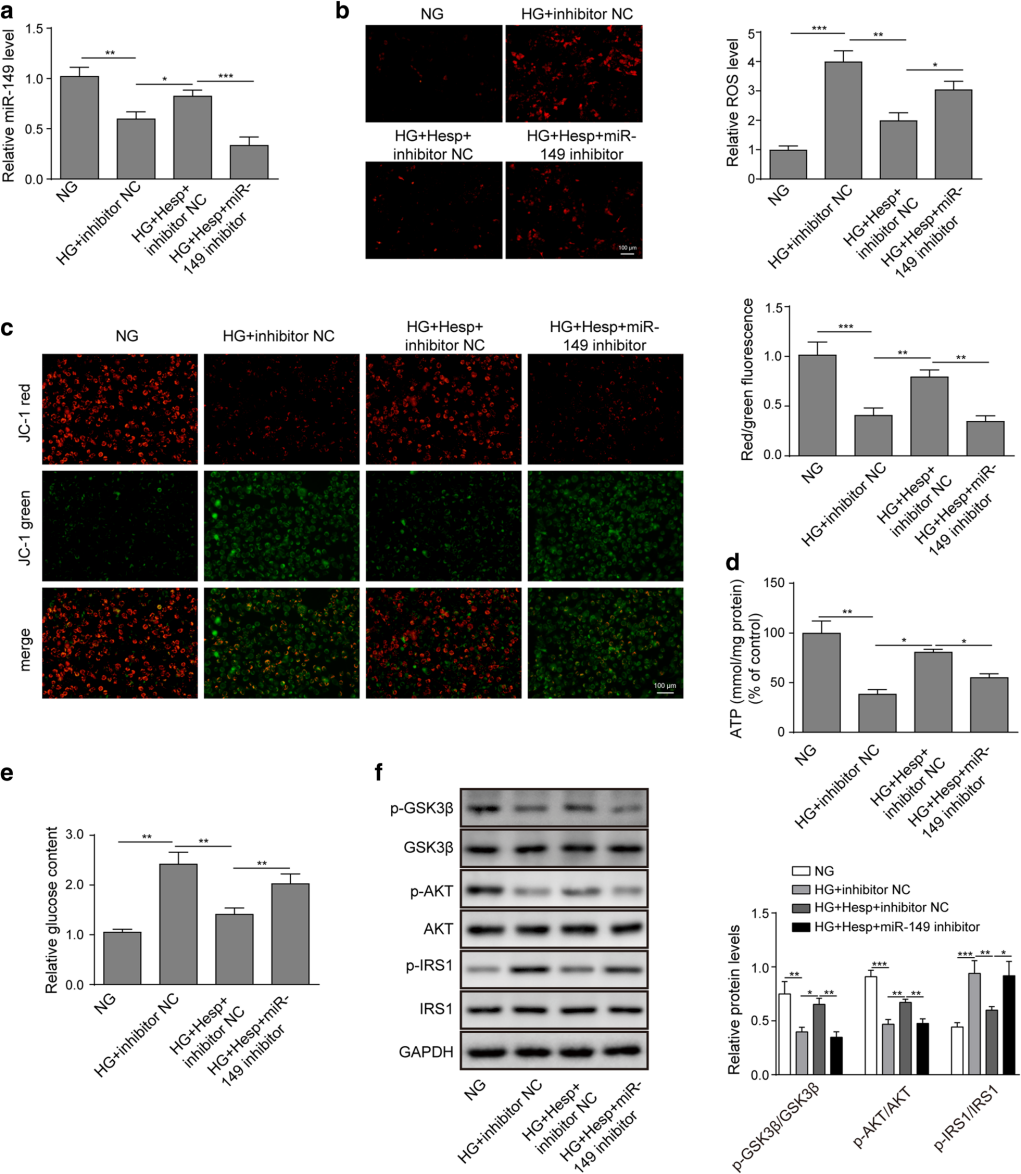
Knockdown of miR-149 partly reversed the biological effects of hesperidin. **a** The transfection efficiency of the miR-149 inhibitor was detected by qRT-PCR. **b** The effect of miR-149 downregulation on hesperidin-mediated ROS production was detected by a fluorescent MitoSox probe. **c** The effect of miR-149 downregulation on the hesperidin-regulated MMP level was determined by JC-1 staining. **d, e** The effects of miR-149 downregulation on hesperidin-regulated ATP and glucose contents were quantified by corresponding detection kits. **f** The effects of miR-149 downregulation on hesperidin-mediated phosphorylation of GSK3β, AKT and IRS1 were quantified by western blotting. All the data were presented as the mean ± S.D. *p < 0.05, **p < 0.01, *** *p* < 0.001

Finally, we investigated whether miR-149 was involved to the biological influences of hesperidin in the HG-induced cells. Figure 5a suggested that the increase of miR-149 expression induced by hesperidin was abolished after transfection with the miR-149 inhibitor. Next, the detections of ROS and MMP showed that the downregulation of miR-149 in HG-treated LO2 cells clearly diminished the decreased ROS production and increased MMP level induced by hesperidin (Fig. 5b, c). Similarly, the hesperidin-mediated the promotion of ATP production was also weakened by miR-149 downregulation (Fig. 5d). Conversely, miR-149 downregulation abated the inhibitory effect of hesperidin on glucose production (Fig. 5e). Furthermore, compared to hesperidin treatment, miR-149 knockdown significantly inhibited the phosphorylation of GSK3β and AKT while increasing the phosphorylation of IRS1 (Fig. 5f). Taken together, the rescue experiments suggested that miR-149 was a downstream functional target of hesperidin in HG-treated LO2 cells.

## Discussion

Oxidative stress is thought to be a key contributor to the onset and progression of mitochondrial dysfunction, insulin resistance and β-cell dysfunction [29]. It has been reported that oxidative stress and mitochondrial dysfunction are common in diabetes [25]. Additionally, the underlying aetiology of insulin resistance in diabetes has been confirmed to correlate with the selective deletion of the IRS1 and IRS2 proteins, the elevated phosphorylation of IRS-1 Ser(307) and the reduced phosphorylation of Akt Ser(473) and GSK-3β Ser(9) [30]. Yan et al. also thought that the activated phosphorylation of AMPK/NOX4/PI3K/AKT/GSK3β promoted hepatic glycogen synthesis and ultimately ameliorating the hepatic insulin resistance in type 2 diabetes, indicating that the disruption of the insulin signalling pathway was correlated to liver insulin resistance [24]. High concentrations of glucose result in an imbalance of the cell redox status in hepatocytes and induce insulin resistance [31]. By referring to a previous study [24, 31–33], we established an oxidative stress model by stimulating LO2 cells with HG to investigate the effects of hesperidin on oxidative stress, mitochondrial function and insulin resistance as well as the underlying mechanisms.

A large numerous of study has reported that hesperidin has analgesic, antihypertensive, diuretic, hypolipidaemic, anti-inflammatory, anti-cancer and antioxidant effects [11, 12]. Mounting studies have demonstrated that hesperidin served multiple protective roles against diabetes progression. For instance, hesperidin upregulated the GLUT2, GLUT4, IRS1 and glucokinase levels and downregulated the hepatic fatty acid oxidation and carnitine palmitoyl transferase activity in type-2 diabetes to improve hyperlipidaemia and hyperglycaemia [34, 35]. Kanwal et al. also reported that hesperidin relieved insulin resistance by inhibiting inflammatory responses [36]. In this study, our study showed that HG treatment successfully exhibited a low antioxidant activity, and accompanied by mitochondrial dysfunction and insulin resistance in LO2 cells, suggesting that the diabetic model of oxidative stress was successfully established in vitro. Moreover, our data demonstrated that hesperidin improved cell viability, oxidative stress, and mitochondrial dysfunction and eventually alleviating the insulin resistance of HG-treated LO2 cells, which may associated with the increase in the phosphorylation of AKT and GSK-3β and a decrease in IRS1.

Increasing numbers of reports have shown that hesperidin exerts protective effects on neuroinflammation and lipid metabolism by regulating miRNA expression, indicating that miRNAs might be a functional target targets of hesperidin [37, 38]. In addition, hesperidin has identified as a DNA hypomethylating agent that modulates gene expression patterns [26, 27]. For instance, hesperidin could upregulate the expression of SFRP2 in adjuvant arthritis rats by reducing DNMT1 [39]. In the present work, our data revealed that hesperidin restored the expression of miR-149 in HG-stimulated LO2 cells by suppressing the DNMT1-mediated methylation of its promoter. Consistently, previous reports have proven that the expression of miR-149 was regulated by CpG methylation, further confirming our results [28, 40]. Additionally, miR-149 was a key modulator of insulin resistance and mitochondrial dysfunction in skeletal muscle [23]. Mohamed et al. showed that miR-149 promoted mitochondrial biogenesis in skeletal muscle by directly inhibiting PARP-2 [41]. Ruan et al. also demonstrated that miR-149 protected pancreatic beta cells from HG-induced apoptosis and ROS generation and elevated cell viability and insulin secretion by targeting BIM [22]. These observations indicated that miR-149 served a protective role in diabetes. Here, our data illustrated that knockdown of miR-149 diminished the regulatory effects of hesperidin on oxidative stress, mitochondrial dysfunction and insulin resistance in HG-treated LO2 cells, indicating that miR-149 was a functional target of hesperidin and plays a beneficial effect in diabetes.

## Conclusions

In conclusion, our findings demonstrated that hesperidin improved oxidative stress, mitochondrial dysfunction and insulin resistance by inhibiting DNMT1-mediated miR-149 silencing. These findings further elucidated the potential therapeutic value of hesperidin in type 2 diabetes.

## Data Availability

The datasets used or analyzed during the current study are available from the corresponding author on reasonable request.

## Abbreviations

HG: High glucose
MMP: Mitochondrial membrane potential
MSP: Methylation-specific PCR
SOD: Superoxide dismutase
ATP: Adenosine triphosphate
GPx: Glutathione peroxidase
ROS: Reactive oxygen species
MDA: Malondialdehyde
miRNA: MicroRNA
DMEM: Dulbecco’s modified Eagle medium
FBS: Foetal bovine serum
qRT-PCR: Quantitative real-time PCR
